# Supplementary material for: Archival skin biopsy specimens as a tool for miRNA-based diagnosis: Technical and post-analytical considerations
Source: Mol Ther Methods Clin Dev. 2023 Sep 16;31:101116. doi: 10.1016/j.omtm.2023.101116 (PMC10550798; doi:10.1016/j.omtm.2023.101116)
Supplement: Document S1. Tables S1, S2, S4, and S5 [file mmc1.pdf]

OMTM, Volume *31*

Supplemental information

Archival skin biopsy specimens as a tool for miRNA-based diagnosis: Technical and post-analytical considerations

Mirna Andelic, Margherita Marchi, Stefania Marcuzzo, Raffaella Lombardi, Catharina G. Faber, Giuseppe Lauria, and Erika Salvi

Table S1. Demographic characteristics of the study groups.

	Patients (n=31)	HC (n=19)
**Female**	15 (48.39%)	9 (47.37%)
**Age at biopsy**	48.9±16.3	44.7±16.9

Age and sex are not statistically significantly different between groups.

Table S2. RNA purity and concentrations values measured by NanoDrop ND-1000 Spectrophotometer.

Sample ID	Phenotype	RNA QC (NanoDrop)	
		c (ng/µl)	Purity (260/280)
1132	Pain	6.5	1.81
2048	Pain	5.02	1.90
2137	Pain	6.06	1.87
2453	Pain	8.09	1.90
2511	HC	6.69	1.80
2614	HC	7.93	1.92
2696	Pain	7.70	1.80
2780	HC	5.0	1.85
2841	Pain	8.50	1.80
2856	Pain	8.20	1.95
2913	Pain	8.16	1.99
2971	Pain	8.65	1.90
3077	HC	13.56	1.82
3105	HC	5.0	1.92
3106	HC	5.3	2.00
3125	HC	5.6	1.85
3258	Pain	7.93	1.80
3269	HC	9.93	1.87
3288	Pain	8.06	1.80
3508	HC	8.5	2.0
874	Pain	8.1	2.00
978	HC	7.7	1.93
HC_1	HC	12.20	1.80
HC_2	HC	7.00	1.92
HC_3	HC	13.50	1.85
HC_4	HC	5.1	1.80
HC_5	HC	5.20	1.80
HC_6	HC	4.85	1.82

Sample ID	Phenotype	RNA QC (NanoDrop)	
		c (ng/µl)	Purity (260/280)
HC_7	HC	4.89	1.80
HC_8	HC	6.40	1.94
HC_9	HC	7.70	1.83
M1	Pain	8.10	1.85
M2	Pain	5.50	1.90
M3	Pain	9.70	1.90
M4	Pain	4.50	1.85
M5	Pain	5.20	1.78
M6	Pain	9.50	1.79
SFN_1	Pain	13.40	1.80
SFN_11	Pain	7.30	2.01
SFN_12	Pain	7.50	1.85
SFN_13	Pain	5.00	1.82
SFN_14	Pain	6.70	1.80
SFN_2	Pain	8.80	1.82
SFN_3	Pain	7.10	1.80
SFN_4	Pain	7.00	2.00
SFN_5	Pain	8.80	1.92
SFN_6	Pain	5.10	1.90
SFN_7	Pain	4.20	1.80
SFN_8	Pain	7.30	1.90
SFN_9	Pain	5.10	1.93

Table S3. Comparison results of Cq values among Automatic, C_{RT} and fixed thresholds. Kruskal-Wallis rank sum test with Dunn's test post-hoc analysis was used for multiple comparisons. Post-hoc analysis results were shown only for significant Kruskal Wallis p-values. Only good amplified miRNAs with call rate>90 and median Cq>32 were compared.

Table S4. Expression stability values of miRNAs in all samples applying BestKeeper, delta Ct, geNorm, Normfinder, and RefFinder algorithms, using C_{RT} threshold. MiRNA expressed in 100% of samples (CR=100) were considered. Abbreviations: SD standard deviation, MV M-value, SV stability value, GM geometric mean.

		BestKeeper		Delta CT		Genorm		Normfinder		RefFinder	
**Card**	**miRNA**	**Rank**	**SD**	**Rank**	**SD**	**Rank**	**MV**	**Rank**	**SV**	**Rank**	**GM**
card A	**miR-200c-002300**	11	1.54	1	1.02	6	0.73	1	0.38	1	2.96
	miR-193b-002367	9	1.45	1	1.02	3	0.61	3	0.42	2	3.83
	[illegible]										
	miR-484-001821	6	1.33	6	1.18	1	0.40	12	0.79	4	4.90
	miR-574-3p-002349	3	1.27	15	1.30	1	0.40	25	0.97	5	6.44
	let-7b-002619	5	1.32	7	1.19	5	0.70	8	0.75	6	6.82
	miR-320-002277	4	1.28	8	1.20	4	0.66	11	0.79	7	7.17
	miR-139-5p-002289	7	1.34	5	1.16	7	0.76	7	0.70	8	7.24
	miR-197-000497	2	1.23	14	1.29	2	0.49	23	0.96	9	7.34
	let-7c-000379	13	1.59	3	1.14	9	0.79	6	0.67	10	7.47
	miR-222-002276	20	1.71	4	1.15	21	1.01	5	0.66	11	10.61
	miR-30c-000419	25	1.84	4	1.15	22	1.02	4	0.65	12	11.25
	miR-191-002299	16	1.62	7	1.19	10	0.81	10	0.76	13	11.52
	let-7d-002283	17	1.64	9	1.23	13	0.88	15	0.82	14	14.68
	U6	1	0.82	26	1.63	36	1.24	38	1.38	15	15.20
	miR-24-000402	21	1.75	8	1.20	25	1.06	9	0.75	16	15.93
	miR-328-000543	14	1.60	13	1.27	14	0.90	20	0.91	17	16.81

		BestKeeper		Delta CT		Genorm		Normfinder		RefFinder	
**Card**	**miRNA**	**Rank**	**SD**	**Rank**	**SD**	**Rank**	**MV**	**Rank**	**SV**	**Rank**	**GM**
	miR-146a-000468	19	1.67	9	1.23	26	1.07	16	0.83	18	18.41
	miR-331-000545	26	1.87	11	1.24	23	1.03	13	0.80	19	19.67
	miR-365-001020	30	1.99	10	1.23	20	0.99	14	0.80	20	19.68
	miR-125a-5p-002198	20	1.71	16	1.32	12	0.86	22	0.95	21	19.71
	miR-342-3p-002260	15	1.61	19	1.37	11	0.84	31	1.07	22	20.06
	miR-211-000514	12	1.55	19	1.37	17	0.94	28	1.03	23	20.64
	miR-145-002278	20	1.71	15	1.30	16	0.93	21	0.92	24	20.84
	miR-150-000473	18	1.66	17	1.35	15	0.91	26	1.01	25	21.01
	miR-223-002295	24	1.80	12	1.25	27	1.08	17	0.83	26	21.82
	miR-99b-000436	28	1.93	13	1.27	19	0.98	19	0.89	27	22.21
	miR-100-000437	29	1.98	12	1.25	24	1.04	18	0.84	28	22.74
	miR-92a-000431	22	1.76	17	1.35	18	0.97	27	1.02	29	24.03
	miR-345-002186	19	1.67	18	1.36	28	1.10	24	0.97	30	25.06
	miR-483-5p-002338	8	1.44	27	1.65	37	1.26	39	1.38	31	26.08
	miR-146b-001097	19	1.67	22	1.43	29	1.11	33	1.10	32	28.43
	miR-126-002228	27	1.88	19	1.37	31	1.14	29	1.03	33	29.96
	miR-186-002285	23	1.77	21	1.42	30	1.13	32	1.08	34	30.43
	miR-203-000507	30	1.99	20	1.38	32	1.16	30	1.05	35	32.19
	miR-19b-000396	31	2.06	23	1.49	33	1.18	34	1.20	36	34.49
	miR-195-000494	32	2.23	24	1.52	34	1.20	35	1.24	37	35.49
	miR-29c-000587	33	2.28	24	1.52	35	1.22	36	1.25	38	36.49
	miR-27a-000408	35	2.37	25	1.61	38	1.28	37	1.37	39	38.23
	miR-125b-000449	36	2.38	28	1.66	39	1.30	40	1.41	40	40.25
	miR-374-000563	34	2.33	29	1.79	40	1.32	41	1.59	41	40.49
card B	**hsa-miR-99b#-002196**	1	0.82	1	1.18	1	0.825	1	0.657	1	1
	hsa-miR-338-5P-002658	2	0.91	4	1.31	1	0.825	4	0.915	2	2.38
	hsa-miR-1285-002822	4	1.04	2	1.2	3	1.049	2	0.668	3	2.83
	hsa-miR-378-002243	5	1.2	3	1.3	2	0.968	3	0.853	4	3.41
	U6	3	0.96	6	1.39	4	1.137	6	1.043	5	4.82
	hsa-miR-1201-002781	6	1.4	5	1.38	5	1.206	5	0.985	6	5.48
	hsa-miR-30d-000420	7	2.11	7	1.72	6	1.353	7	1.501	7	7

Table S5. Stability ranking obtained with the recommended comprehensive analysis from RefFinder, applying different threshold settings. MiRNA expressed in 100% of samples (CR=100) were considered.

	Pool A			Pool B		
**Ranking**	**Fixed**	**Automatic**	**C_{RT}**	**Fixed**	**Automatic**	**C_{RT}**
**1**	**miR-193b-002367**	**miR-200c-002300**	**miR-200c-002300**	**U6-snRNA-001973**	**miR-99b#-002196**	**miR-99b#-002196**
**2**	miR-200c-002300	miR-200b-002251	miR-193b-002367	miR-1285-002822	miR-1285-002822	miR-338-5P-002658
**3**	let-7b-002619	let-7b-002619	miR-200b-002251	miR-99b#-002196	miR-338-5P-002658	miR-1285-002822
**4**	miR-320-002277	miR-193b-002367	miR-484-001821	miR-338-5P-002658	miR-378-002243	miR-378-002243
**5**	let-7c-000379	miR-484-001821	miR-574-3p-002349	miR-378-002243	U6-snRNA-001973	U6-snRNA-001973
**6**	miR-200b-002251	miR-574-3p-002349	let-7b-002619	miR-1201-002781	miR-1201-002781	miR-1201-002781
**7**	miR-191-002299	miR-197-000497	miR-320-002277		miR-30d-000420	miR-30d-000420
**8**	miR-328-000543	miR-320-002277	miR-139-5p-002289			
**9**	miR-222-002276	let-7c-000379	miR-197-000497			
**10**	miR-92a-000431	miR-222-002276	let-7c-000379			
**11**	miR-484-001821	miR-30c-000419	miR-222-002276			
**12**	let-7d-002283	miR-139-5p-002289	miR-30c-000419			
**13**	miR-139-5p-002289	miR-191-002299	miR-191-002299			
**14**	miR-30c-000419	miR-328-000543	let-7d-002283			
**15**	miR-331-000545	miR-24-000402	U6-snRNA-001973			
**16**	U6-snRNA-001973	miR-331-000545	miR-24-000402			
**17**	miR-211-000514	let-7d-002283	miR-328-000543			
**18**	miR-24-000402	U6-snRNA-001973	miR-146a-000468			
**19**	miR-146a-000468	miR-146a-000468	miR-331-000545			
**20**	miR-574-3p-002349	miR-223-002295	miR-365-001020			
**21**	miR-197-000497	miR-99b-000436	miR-125a-5p-002198			
**22**	miR-223-002295	miR-365-001020	miR-342-3p-002260			
**23**	miR-365-001020	miR-100-000437	miR-211-000514			
**24**	miR-342-3p-002260	miR-211-000514	miR-145-002278			
**25**	miR-99b-000436	miR-345-002186	miR-150-000473			
**26**	miR-483-5p-002338	miR-145-002278	miR-223-002295			
**27**	miR-145-002278	miR-125a-5p-002198	miR-99b-000436			
**28**	miR-125a-5p-002198	miR-92a-000431	miR-100-000437			
**29**	miR-146b-001097	miR-150-000473	miR-92a-000431			
**30**	miR-126-002228	miR-146b-001097	miR-345-002186			
**31**	miR-186-002285	miR-483-5p-002338	miR-483-5p-002338			
**32**	miR-195-000494	miR-342-3p-002260	miR-146b-001097			

	Pool A			Pool B		
**Ranking**	**Fixed**	**Automatic**	**C_{RT}**	**Fixed**	**Automatic**	**C_{RT}**
**33**	miR-150-000473	miR-186-002285	miR-126-002228			
**34**	miR-345-002186	miR-126-002228	miR-186-002285			
**35**	miR-100-000437	miR-203-000507	miR-203-000507			
**36**	miR-19b-000396	miR-195-000494	miR-19b-000396			
**37**	miR-203-000507	miR-19b-000396	miR-195-000494			
**38**	miR-29c-000587	miR-19a-000395	miR-29c-000587			
**39**	miR-374-000563	miR-29c-000587	miR-27a-000408			
**40**		miR-27a-000408	miR-125b-000449			
**41**		miR-125b-000449	miR-374-000563			
**42**		miR-374-000563				

Table S6. miRNA relative expression results comparing painful neuropathic against control group, considering C_{RT}, baseline automatic and manually set datasets. miRNAs are ranked for C_{RT} P-value. The comparisons are made applying Wilcoxon rank sum test. For C_{RT} and baseline automatic, normalization was done using miR-200c-002300 (pool A) and miR-99b#-002196 (pool B) whereas for manually set threshold, hsa-miR-193b-002367 (poolA) and U6-snRNA-001973 (poolB) were used. We compared quality-controlled miRNAs with call rate>90 and median Cq>32, in common among the datasets.

Table S7. List of miRNAs expressed in human epidermis from fixed skin biopsy in 19 healthy controls.

Table S8. ClueGO Enrichment analysis results. Molecular functions (MF) and biological processes (BP) inferred from the list of epidermal miRNAs-targets are listed. Bonferroni Pvalue: p value of the GO term after Bonferroni correction. % Associated Genes: percentage of genes found from the total number of associated genes. N Genes: Number of genes from our list of miRNA-targets associated with the GO term. Associated genes are represented from among those associated with GO terms.
