# Supplementary material for: Archival skin biopsy specimens as a tool for miRNA-based diagnosis: Technical and post-analytical considerations
Source: Mol Ther Methods Clin Dev. 2023 Sep 16;31:101116. doi: 10.1016/j.omtm.2023.101116 (PMC10550798; doi:10.1016/j.omtm.2023.101116)
Supplement: Document S2. Article plus supplemental information [file mmc3.pdf]

Molecular Therapy
Methods & Clinical Development
Original Article

Archival skin biopsy specimens as a tool for miRNA-based diagnosis: Technical and post-analytical considerations

Mirna Andelic,[1,2] Margherita Marchi,[1] Stefania Marcuzzo,[3] Raffaella Lombardi,[1] Catharina G. Faber,[4] Giuseppe Lauria,[1,5] and Erika Salvi[1,6]

[1]Neuroalgology Unit, Fondazione IRCCS Istituto Neurologico Carlo Besta, 20133 Milan, Italy; [2]School of Mental Health and Neuroscience, Maastricht University Medical Centre+, P.O. Box 5800, 6202 AZ Maastricht, the Netherlands; [3]Neuroimmunology and Neuromuscular Diseases Unit, Fondazione IRCCS Istituto Neurologico Carlo Besta, 20133 Milan, Italy; [4]Department of Neurology and School for Mental Health and Neuroscience, Maastricht University Medical Centre+, P.O. Box 5800, 6202 AZ Maastricht, the Netherlands; [5]Department of Medical Biotechnology and Translational Medicine, University of Milan, 20133 Milan, Italy; [6]Data Science Center, Fondazione IRCCS Istituto Neurologico Carlo Besta, 20133 Milan, Italy

Archived specimens, taken by standardized procedures in clinical practice, represent a valuable resource in translational medicine. Their use in retrospective molecular-based studies could provide disease and therapy predictors. Microfluidic array is a user-friendly and cost-effective method allowing profiling of hundreds of microRNAs (miRNAs) from a low amount of RNA. However, even though tissue miRNAs may include potentially robust biomarkers, non-uniformed post-analytical pipelines could hinder translation into clinics. In this study, epidermal RNA from archival skin biopsy specimens was isolated from patients with peripheral neuropathy and healthy individuals. Unbiased miRNA profiling was performed using RT-qPCR-based microfluidic array. We demonstrated that RNA obtained from archival tissue is appropriate for miRNA profiling, providing evidence that different practices in threshold selection could significantly influence the final results. We showed the utility of software-based quality control for amplification curves. We revealed that selection of the most stable reference and the calculation of geometric mean are suitable when utilizing microfluidic arrays without known references. By applying appropriate post-analytical settings, we obtained miRNA profile of human epidermis associated with biological processes and a list of suitable references. Our results, which outline technical and post-analytical considerations, support the broad use of archived specimens for miRNA analysis to unravel disease-specific molecular signatures.

INTRODUCTION

Discoveries of microRNA (miRNA)-driven mechanisms of post-transcriptional gene regulation have resulted with identification of molecular signatures associated with specific patient characteristics and enabled their entrance into the clinical setting as powerful disease-specific biomarkers and potential pharmaceutical targets.[1–4] This research field is particularly advanced in cancer and is associated with access to fresh surgical tissues required for molecular profiling, while in a plethora of clinical fields, neurology included, specific studies need to be designed and specimens taken exclusively for study purposes. However, efforts have been made to assess archived specimens, taken for diagnostic purposes, and their quality and utility for molecular profiling,[5–8] which paved new avenues for molecular-based retrospective studies on archived specimens taken for diagnostic purposes or in clinical trials.

Small fiber neuropathy (SFN) is a disease of the sensory system, affecting mainly unmyelinated and thinly myelinated nerve fibers, and it is often accompanied by neuropathic pain.[9–12] For the past 30 years, evaluation of cutaneous innervation is one of the few diagnostic tests used for establishing the diagnosis of SFN.[12] In the pain clinics, skin biopsy tissues are routinely collected and analyzed to aid treatment selection or as the inclusion criteria for novel pharmacological trials, making available valuable tissue biobanks. Molecular signatures of the targeted tissue, such as skin biopsy in the case of SFN, associated with trial outcomes, could provide novel cues and potentially increase rate of responders by highlighting predictors of treatment response in clinical practice.[13,14] miRNAs could represent robust tissue biomarkers and advance pain research and patient management.

Even though potential miRNA candidates have been identified, conflicting findings across studies and poor clinical translation remain.[15] The motives could be various, from the features of the clinical cohorts investigated, to different post-analytical methodological approaches. The latter has been emphasized by other researchers who advocated for the standardization of miRNA profiling settings

Received 27 June 2023; accepted 13 September 2023;
https://doi.org/10.1016/j.omtm.2023.101116.

Correspondence: Erika Salvi, PhD, Data Science Center and Neuroalgology Unit, Fondazione IRCCS Istituto Neurologico Carlo Besta, 20133 Milan, Italy.
E-mail: erika.salvi@istituto-besta.it

This is an open access article under the CC BY license (http://creativecommons.org/licenses/by/4.0/).

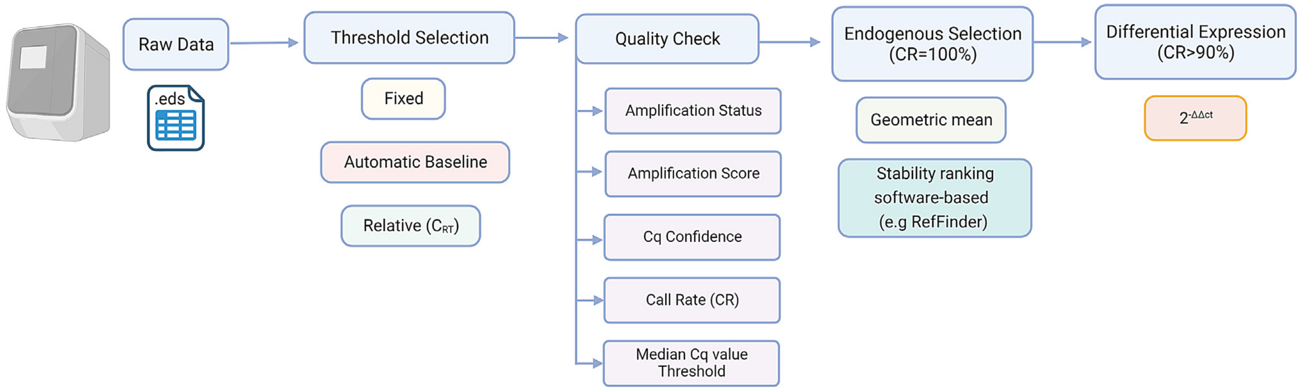

Figure 1. Flowchart of post-analytical settings

when utilizing microarrays to achieve inter-laboratory agreement and reproducibility.[15,16]

Currently, one of the most widely and cost-efficient TaqMan-based methodologies for low-quantity starting material miRNA profiling is the RT-qPCR microfluidic array. This is a highly sensitive and accurate technique enabling the identification of hundreds of miRNAs from samples yielding a low quantity of RNA.[17] However, post-analytical settings can affect results and must be considered. First, when generating data from the RT-qPCR systems, the threshold needs to be determined to generate the quantification cycle (Cq) for each amplified curve.[18,19] Several threshold algorithms could be selected, considering the number of analyzed samples and targets and should be determined according to the guidelines for the specific array and reported in the publication for data reproducibility. Second, relative expression analysis should be applied only to the targets with reliable amplification curves. RT-qPCR instruments are supplied with running and analysis software, that allow users to set the threshold and provide scores for automatic quality check (QC) filtering of amplification curves. When QC is not applied, the use of unreliable curves for further analysis could result in false positive data production. Finally, the accurate determination of the relative levels of miRNAs requires normalization using a reference or endogenous control that should ideally be constant, stable, unregulated, and unaffected by experimental conditions. For this reason, it should be selected considering its quality and stability in the sample of interest and studied groups.[20] Variables that can influence stability are affected by the experimental settings, the origin of the tissue sample and its heterogeneity, the quantity and stability in different specimens, and the sample handling and storage.[21–23]

With this study, we aimed to provide a post-analytical pipeline for microfluidic array-based miRNA profiling in archived specimens, identifying several key steps that need to be considered when analyzing hundreds of miRNA amplifications in microfluidic system. We compared analysis using the post-analytical settings most widely used in literature and observed significant influence on final results, applied in the illustrative experiment on RNA isolated from fixed skin biopsy tissue, derived from healthy controls and patients suffering from neuropathic pain caused by different etiologies. Therefore, this analysis was not aimed at discovering differentially expressed miRNAs related to the pathology but provides indications for critical post-analytical approaches for threshold setting, quality control, and endogenous selection. Finally, to improve the knowledge on the molecular mechanisms that regulate epidermis, we provided an miRNA profile and a list of stable reference miRNAs that could serve as references in single-assay-based future studies.

RESULTS

We used RNA samples extracted from the epidermal part of skin biopsy from 31 neuropathic pain patients versus 19 healthy controls as a showcase.

To contribute toward more unified settings, we described and compared (1) the most widely used thresholds for Cq generation; (2) amplification curve QC options generated by the RT-qPCR instrument, useful for the identification of reliable amplifications; and (3) tools for endogenous miRNA selection (Figure 1). Applying the post-analytical settings, we provided a comprehensive overview of human epidermal miRNA profile.

Threshold algorithms setting and amplification curve QC

First, we analyzed the raw data by setting all three threshold algorithms: automatic baseline, manual baseline fixed at 0.2, and relative threshold (C_{RT}) (Figure 2A). The manual fixed is set by the user in the linear portion of the amplification curves (default = 0.2.). The automatic threshold is automatically determined by the instrument, one for each miRNA of the experiment. For the relative threshold (C_{RT}), using an empirically predetermined reference fluorescence value and a proprietary algorithm, a common point on the reaction efficiency curve is identified and used to map back to the original amplification curve. The C_{RT} is the method of choice, recommended by ThermoFisher, when dealing with hundreds of targets (Figure 2A).[24]

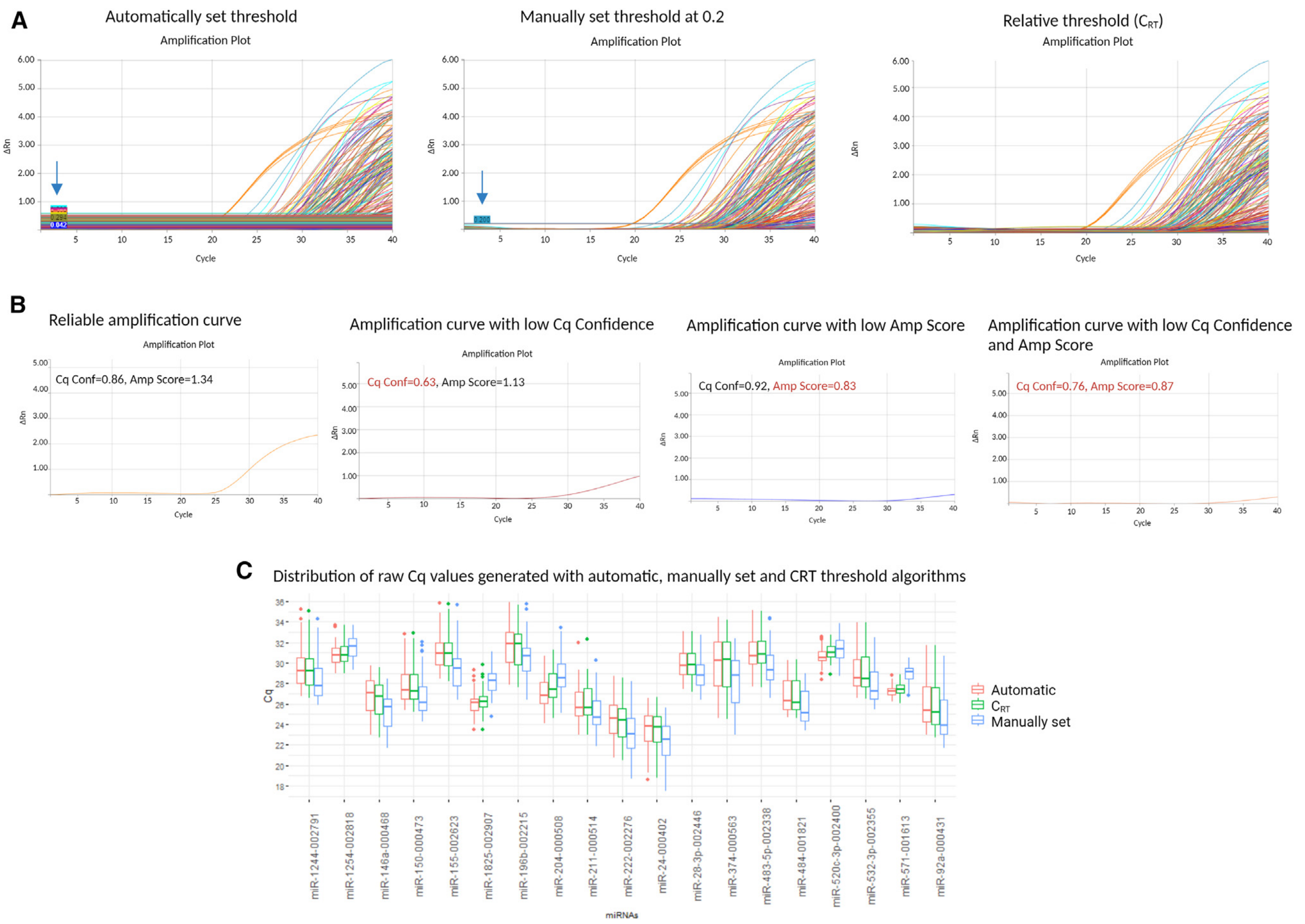

Figure 2. Threshold algorithm settings and amplification curve quality checks

(A) Threshold algorithms. Amplification plots represent the automatically set threshold where each curve has its own threshold (blue arrow), the manually set threshold at 0.2 where the Ct for all curves is calculated considering the single threshold (blue arrow), the relative threshold (C_{RT}). The representative plot originates from a single card of healthy control. (B) Amplification curve quality check. The plots show different amplification curves according to quality parameters (Amp Score, Cq conf). The reliable amplification curves have Amp Score above 1 and Cq Confidence above 0.8. The unreliable curves are the ones not meeting these criteria. The representative curves originate from the single card of healthy controls. (C) Distribution of raw Cq values generated with manually fixed, automatic, and C_{RT} threshold algorithms. Bar graph indicates the Cq values obtained after the three thresholds selection (automatic, C_{RT}, or manually set at 0.2) for miRNAs with Kruskal p values <0.01. Only good-quality miRNAs with call rate >90 and median Cq >32 were compared.

Since only reliable amplification curves should be used for further analysis, we evaluated the aspects of amplified curves, comparing the QC scores. The major advantage of this QC is the automatic filtering when handling hundreds of amplifications in a single run, where the visual inspection is impractical and very time-consuming. Considering AmpScore >1 and CqConf >0.8 as thresholds for good amplification, we checked the curves with a different type of scores (Figure 2B). With AmpScore >1 and CqConf > 0.8 we observed a high linear region rise, confirming the association with strong amplification. When one, or both, parameters are under cutoff, the curves do not have a linear region resulting in non-reliable amplification (Figure 2B). Setting this threshold reliably substitutes time-consuming manual inspection of the single amplification curve helping to resolve ambivalent data. After the QC, the miRNAs with good-quality values (AmpScore >1, CqConf >0.8) are 551 (73.1%) for C_{RT}, 547 (72.5%) for automatic, and 547 (72.5%) for the manually set threshold.

Comparison of threshold settings

For all three threshold algorithms, we compared the number of amplified miRNAs and the raw Cq values, after selecting only curves with good-QC values (AmpScore >1, CqConf >0.8). We considered only miRNAs that are expressed in at least 90% of samples (call rate ≥90%). Considering different call rate categories (90-92-94-96-98-100%), we noted that there was no great difference among the three thresholds considering the cumulative number of expressed miRNAs. The percentage of miRNAs with reliable amplification in 100% of samples in C_{RT} was 8.7%, in automatic threshold 9.0%, while slightly fewer miRNAs (8.2%) were detected when applying manually fixed threshold (Table 1).

Table 1. Call rate of miRNAs with reliable amplification

	Relative threshold (C_{RT})		Automatic threshold		Manually set threshold at 0.2	
Call rate	Number	Percent	Number	Percent	Number	Percent
100%	48	8.7%	49	9.0%	45	8.2%
98%	76	13.8%	75	13.7%	71	13.0%
96%	94	17.1%	94	17.2%	81	14.8%
94%	103	18.7%	104	19.0%	101	18.5%
92%	113	20.5%	113	20.7%	111	20.3%
90%	121	22.0%	120	21.9%	119	21.8%
Total miRNAs	551		547		547	

Cumulative numbers and percentages of miRNAs for each call rate category considering different threshold algorithms. Call rate is the percentage of samples with amplification data for the specific miRNA.

We considered an extra level of quality filter, by excluding miRNAs with a median expression of Cq >32: percentage of barely expressed miRNAs in C_{RT} was 5% (n = 6 miRNAs), in automatic threshold 3.4% (n = 4), while we observed the 1.7% (n = 2) when applying a manually fixed threshold.

Indeed, the previous filters based on amplification curve quality and call rate threshold (90%) ensure that only reliably amplified miRNAs will be included in downstream analysis (N = 115 for C_{RT}, N = 116 for automatic and N = 117 for manually set threshold).

To inspect differences among the three threshold algorithms, we compared the raw Cq values of miRNAs that passed quality filters. We observed that the distribution of Cq values generated with the manually fixed threshold at 0.2 was significantly different when compared with the values generated with other two thresholds (automatic and C_{RT}). Kruskal-Wallis rank-sum test with Dunn's test post hoc analysis was used for multiple comparisons (Table S3). In Figure 2C, we reported as an example the distribution of Cq values for the different threshold algorithms for the miRNAs with Kruskal p value <0.01, showing a different distribution, particularly in the manually fixed one.

Reference miRNA selection in human epidermis

Many variables can influence the endogenous selection such as the experimental settings, the origin of the tissue sample and its heterogeneity, the quantity and stability in different specimens, and the sample handling and storage. Thus, the stability of reference miRNAs needs to be checked in each experimental condition. To address the variability issue in the presented experimental system, we followed two main strategies using as input only the miRNAs expressed in all samples (call rate 100%): (1) identification of stable endogenous miRNAs according to stability ranks and (2) the calculation geometric mean by card, as a normalization factor. To evaluate the expression stability of reference miRNAs, different statistical algorithms such as BestKeeper, delta Ct, geNorm, Normfinder, and RefFinder, were employed. Given that the suggested endogenous controls indicated by ThermoFisher (U6, RNU48, RNU44) were not stable after stability evaluation or were not expressed in all samples, we considered miRNAs that were top ranked by the major part of the applied algorithms (Table S4). In pool A, Delta Ct, NormFinder, and RefFinder equally ranked miR-200c-002300 (hsa-miR-200c-3p) as the most stable miRNA, whereas BestKeeper and GeNorm showed inverted places in the rank of miRNAs not concordant within each other. In pool B, miR-99b#-002196 (hsa-miR-99b-3p) emerged as the most suitable normalization control according to all tested algorithms. The same endogenous were selected for C_{RT} and automatic baseline threshold whereas hsa-miR-193b-002367 (poolA) and U6-snRNA-001973 (poolB) resulted in more stability with manually fixed threshold selection (Tables S4 and S5).

As a second normalization approach, the geometric mean of Cq values was calculated in both pools averaging all miRNAs.

The results obtained with two normalization approaches gave consistent results when the expression values of selected endogenous controls and the global geometric mean of the entire plate were compared. Figure 3A shows high correlation comparing geometric mean values with the expression values of best stable miRNAs, hsa-miR-200c-3p (R = 0.98, p value = 2.2e-16) and hsa-miR-99b-3p (R = 0.84, p value = 1.4e-14).

Additionally, we evaluated the distribution of the best-suited reference miRNAs versus global normalization via geometric mean, comparing the disease and the healthy control groups (Figure 3B). In cards B, the global values of geometric mean do not appear to be stable among the studied groups (p = 0.041). The recommendation is to evaluate the averaged values of geometric mean by comparing the inter- and intragroup values. On the contrary, we demonstrated that the RefFinder was able to calculate and rank the comprehensive stability values by considering intragroup and intergroup variation, to select candidate reference miRNAs.

Furthermore, applied post-analytical pipeline allowed to rank and identify reference miRNA candidates in human skin epidermis (Table S5). The top 10 most stable miRNAs, whose Cq distribution is shown in Figure 3C, could be considered as first-choice references in other TaqMan-based assays, using this type of tissue.

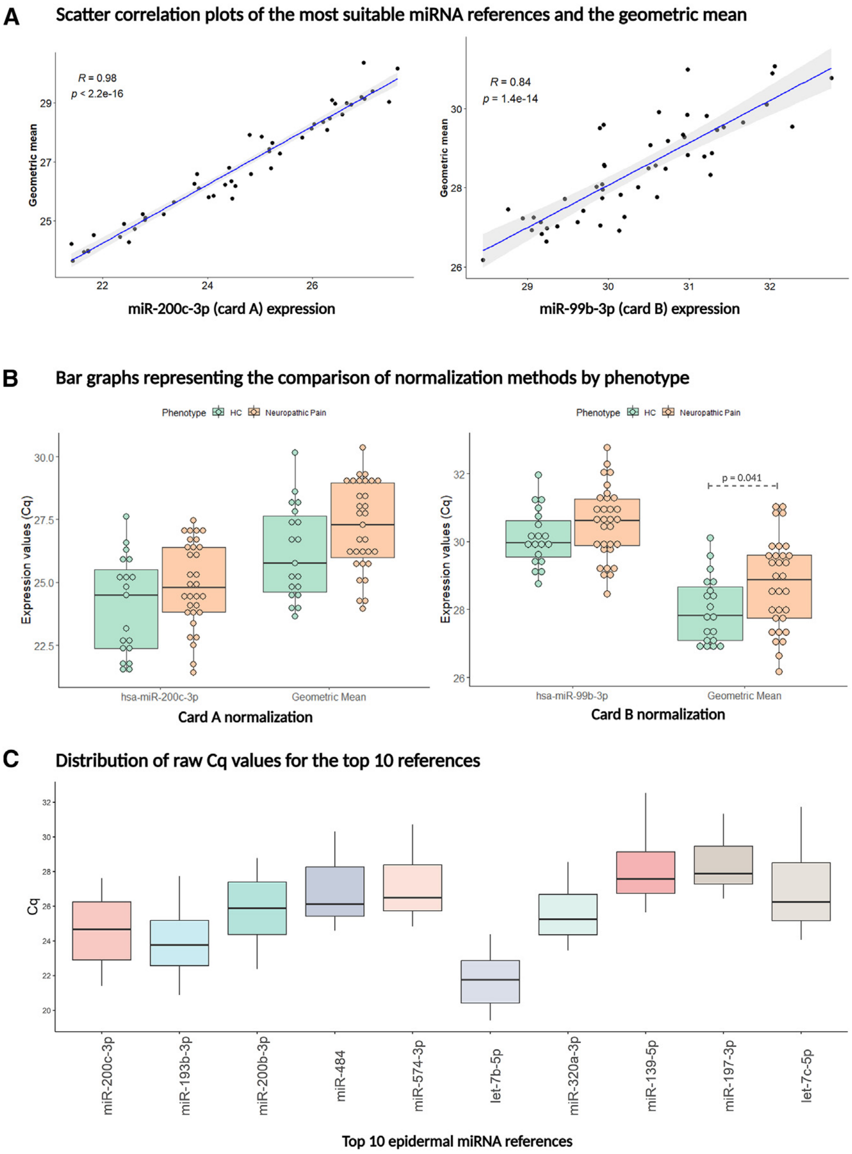

Figure 3. Human epidermal miRNA references

(A) Scatter correlation plots comparing the most suitable miRNA references and the geometric mean. The plot reports the correlation between raw Cq values of hsa-miR-200c-3p as the most suitable miRNA reference for pool A and hsa-miR-99b-3p for pool B with geometric mean of the entire plates. Geometric mean has been calculated on Cq values. Pearson coefficient and p value are shown in the graphs. (B) Bar graphs representing the comparison of normalization methods by phenotype. The comparisons of disease (orange) and healthy control (green) groups are made applying the Wilcoxon rank-sum test. Significant p values are shown in the graphs. Geometric mean has been calculated on Cq values. (C) Boxplot of Cq distribution for the top 10 epidermal miRNA references, ranked according to their stability in fixed human skin epidermis.

Relative expression analysis

The differential expression (DE) of miRNAs was calculated using the relative quantification (RQ) method applying the $2^{-\Delta\Delta ct}$ approach[18] with healthy controls used as the reference group (Table S6). To test the effect of post-analytical settings on results, the relative expression analysis was applied to datasets generated with all three thresholds: manually fixed, automatic, and C_{RT} (Table S6). The relationship among results, obtained with different settings, was investigated with correlation matrix considering fold change (FC) and p value (Figure 4). The analysis shows that values obtained with C_{RT} and automatic threshold highly correlate (FC, R = 0.92, Figure 4A; p value, R = 0.93; Figure 4B), even though they do not completely overlap considering the rank of best DE miRNAs (Table S6). On the contrary, data generated with manually fixed threshold are significantly different from the other two settings, particularly in terms of statistical significance (R = 0.26–0.28, Figure 4B).

miRNAs expressed in human epidermis

A secondary objective of this study was to evaluate if stored skin biopsy samples could be used for miRNA profiling and allow retrospective analysis applying the latest innovative approaches, particularly in the area of neuropathic pain and dermatological conditions for which these samples are taken as part of the standard diagnostic procedure.[10,12,25,26]

To provide a comprehensive list of miRNAs expressed in human epidermis from fixed skin biopsy, we considered only data from healthy subjects. Out of the 754 miRNAs included in the cards A + B, 469 (62%) showed detectable expression in skin epidermis and passed quality filters. The details are reported in Table S7.

For target gene analysis of miRNAs present in at least 95% of healthy subjects, an in silico prediction analysis of experimentally validated miRNA-gene interactions was performed by DIANA-TarBase, selecting 484 target genes reported in skin. We performed an over-representation analysis of Gene Ontology (GO) Biological Processes and Molecular Functions starting from the list of miRNA targets. The GO enriched terms are represented in Figure 5 and listed in Table S8.

DISCUSSION

Archived specimens represent a unique snapshot of the patient's biology in a specific time period, associated with clinical characteristics collected for diagnostic purposes. One of the most valuable characteristics of archived specimens is their large number. The ability to study molecular changes from large patient cohorts within the same pathology and correlate results with the clinical signs of that specific moment

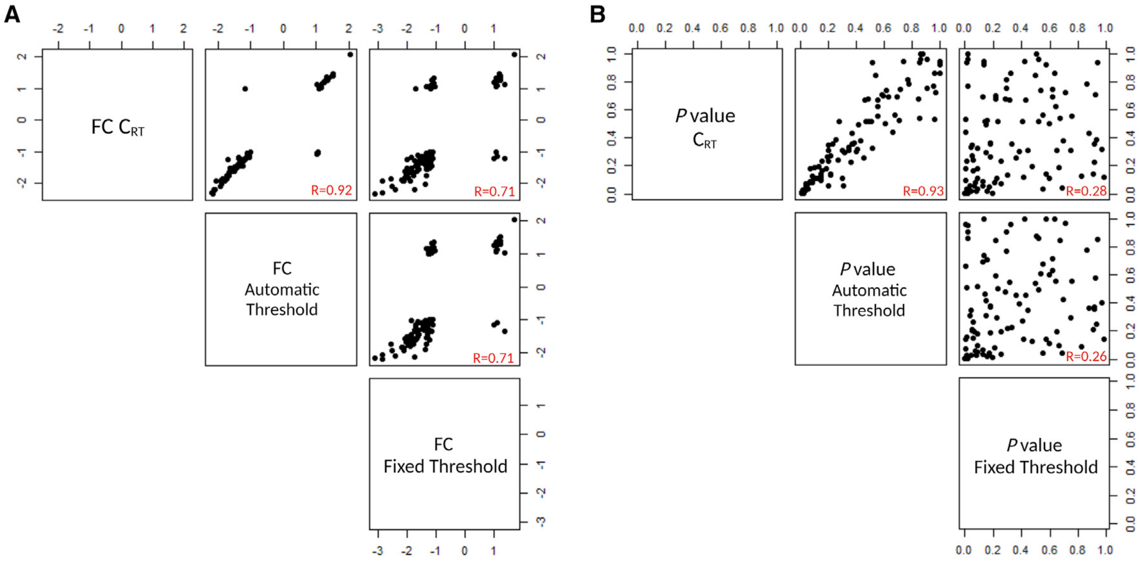

Figure 4. Comparison of the results obtained using different thresholds

Scatterplot matrix for FC (A) and p value (B) of differential expression analysis comparing C_{RT}, automatic, and manually fixed threshold settings. Pearson correlation coefficients (R) are reported. FC, fold change.

is a game changer in biomarker discovery. Many advanced techniques nowadays are optimized for archived specimens,[5–8,27,28] nonetheless due to their user-friendliness and cost-effectiveness, RT-qPCR-based approaches are the most widely used. RT-qPCR-based microfluidic cards are user-friendly nanoliter-scaled techniques that enable the detection of hundreds of miRNAs simultaneously, starting from as little as 1 pg of total RNA. This method is easily applied in a standard molecular laboratory, equipped with either a fast or standard real-time PCR system. Alternative miRNA profiling methods are available, such as the SYBR green approach. However, TaqMan technology has the advantage of higher specificity. Unlike SYBR-green-based approaches, which utilize a non-specific intercalating dye for target detection, TaqMan Advanced miRNA assays utilize a TaqMan MGB probe that is specific only for the miRNA of interest. Another shortcoming of SYBR-based approaches is sensitivity, especially in challenging sample types such as fixed skin biopsies. Microfluidic cards, as a nanoliter-scaled technique, save not only 75% reagents, but also sample volume compared with standard 96-well plates needed for SYBR green approach. Since microfluidic technology differs greatly when compared with single-assay approach, considering the increased number of targets and sample volume, careful data elaboration is required. Thus, as with every qPCR-based technique, a rigorous procedure must be followed when planning and performing experiments to allow inter- and intra-laboratory reproducibility.[29,30]

To contribute toward more unified standards for microfluidic RT-qPCR-based miRNA profiling, we focused on reviewing and comparing post-analytical settings that we found highly heterogeneous in published literature. As a showcase, an miRNA profiling experiment on RNA extracted from fixed skin biopsy samples was used to provide the stepwise post-analytical procedure.

In this study, we showed that stored skin biopsy samples, used in standard diagnostic procedures, could be further used for miRNA profiling, yielding an overview of miRNAs expressed in the epidermis (Table S7), improved knowledge of biological processes that regulate this tissue (Table S8), and a list of possible reference miRNAs that could serve as a first-choice normalization for future relative expression experiments in this tissue (Figure 3; Table S5).

Selection of a robust normalization strategy is mandatory; when utilizing microfluidic RT-qPCR arrays containing hundreds of probes in a sample without known references, two strategies have been proven to be suitable and coherent: (1) selection of the most stable reference miRNA based on stability ranking, and (2) calculation of global geometric mean after evaluation of intergroup stability. We observed a low stability ranking of U6, making it not suitable as an endogenous control for normalizing RQ data in epidermal tissue. This could be due to structural differences between snRNAs made of 150 nucleotides compared with length of miRNAs ranging from 20 to 24 nucleotides. Other authors supported this hypothesis with numerous miRNA profiling studies providing tissue-specific miRNA reference candidates.[31–33]

We showed that the initial software-generated amplification curve QC is helpful to replace the time-consuming manual inspection, in this type of experiment, to discard amplification curves with low quality. In discovery experiments it is important to identify robust molecules, therefore another visual inspection is recommended once the candidate molecule is identified after ddCt analysis, to make sure that the amplification satisfies all standards.[34,35] When analyzing the RT-qPCR microfluidic card studies from January 2019 until September 2022, we noted that QC filtering was never mentioned,

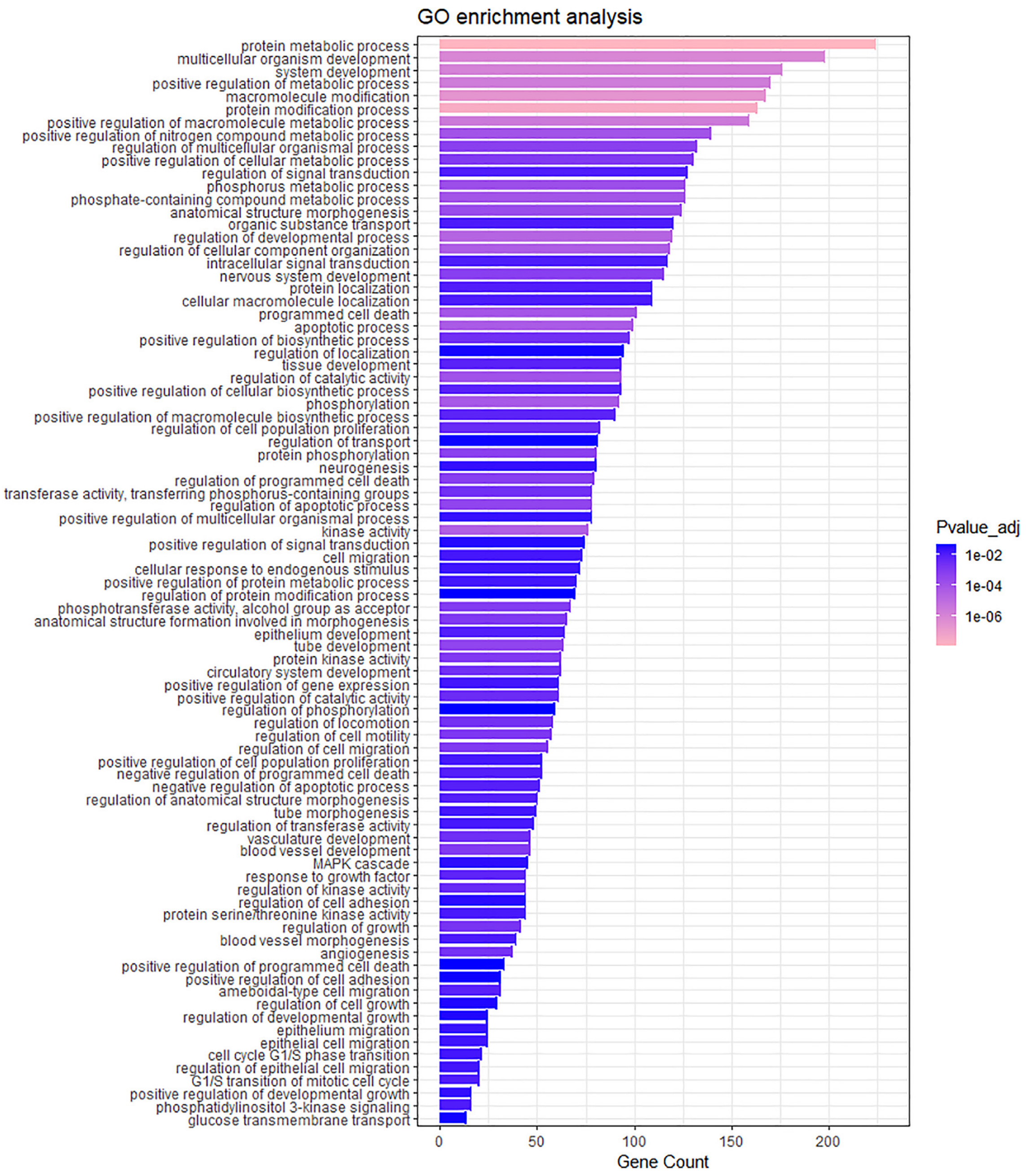

Figure 5. Bar plot of enriched biological processes (BP) and molecular functions (MF) terms

Graph of over-representation analysis results based on the enrichment right-sided hypergeometric test of GO terms starting from the list of miRNA targets expressed in skin. Gene counts are depicted as bar length. Colors refer to the Bonferroni adjusted p value.

leaving the doubt if even applied. The latter could result in false positive data production, biased using unreliable amplification curves, thus affecting data reproducibility.

As an additional quality filter, many manufacturers suggest setting the cutoff value for Cq at the 32nd cycle. However, by fixing a cutoff value for Cq, we risk omitting low-expressed miRNAs in downstream analysis, even if they may have biological relevance in distinguishing patients from healthy controls. An appropriate solution to limit this risk is to consider as a cutoff value the median miRNA Cq.

With this work, we revealed how threshold selection has a significant impact on results, particularly when using a fixed threshold (Figure 5).

A key attribute of the real-time qPCR-based study is a good amplification specificity and efficiency. In this study, we have not evaluated these parameters. However, previously published works experimentally showed that miRNA TaqMan assays are specific for mature miRNAs and able to discriminate miRNAs even if their sequence uniqueness is based only on 1 nucleotide change.[36,37] Furthermore, the use of stem-loop technology is the most efficient technology on the market.[37] Microfluidic array used in this study contained manufacturer validated miRNA primers.[38] Archived specimens represent valuable resources in clinical research, which is proven by the fact that novel molecular techniques are optimized considering the limitations of this type of sample.[5,7,27,28,39] RNA in fixed and archived samples is usually highly fragmented requiring preliminary RNA quality and integrity analysis before performing expensive and highly sensitive experiments.[27,40] However, previously published experiments highlighted that miRNAs are usually not affected by fragmentation, being themselves around 20 nucleotide-long fragments. Furthermore, their short sequence makes them more stable and robust over time.[39,41] To date, techniques suitable to evaluate the quality and quantity of miRNAs are limited and when starting new experiments, researchers are left with trial-and-error methods. Here, we showed that RNA extracted from archived fixed specimens represents a good resource to quantify miRNAs utilizing the TaqMan microfluidic array approach.

A potential limitation of the study is the use of a case sample composed of patients with different etiologies, which prevents the identification of disease-specific miRNA candidates. However, considering that the primary aim of this study was to probe post-analytical settings and draw meaningful conclusions regarding the analysis pipeline, we included all the available RNA samples derived from the same tissue type and collection period. For this reason, we showed biological processes only in healthy controls, since the use of a not homogeneous phenotype could lead to the identification of misleading pathways related to DE miRNAs.

We emphasize that the consideration of this overview could provide more uniform, comparable, and reliable results in microfluidic array RT-qPCR-based investigations.

MATERIALS AND METHODS

Study cohort

We performed an unbiased miRNA profiling of 754 miRNAs in skin biopsies from 31 patients with painful peripheral neuropathy and 19 healthy controls recruited in Fondazione IRCCS Istituto Neurologico “Carlo Besta” of Milan, Italy (FINCB) and Maastricht University Medical Center+ (Maastricht UMC+), Maastricht, The Netherlands (Table S1). The study was approved by the local ethical committee (November 7, 2018, approval no. 56) of the Fondazione IRCCS Istituto Neurologico “Carlo Besta” of Milan, under the PAIN-net project (grant agreement number 721841). All experiments were performed in accordance with relevant guidelines and regulations. Written informed consent was obtained from each participant.

Skin biopsy

All skin biopsies were collected at the distal site of the leg, within the territory of the sural nerve, during the neurological visit according to standard procedures using a disposable punch with 3-mm diameter.[42] The biopsies were handled following the standard diagnostic procedure, starting with fixation in 2% periodate-lysine-paraformaldehyde overnight, serial sectioning in 50-mm sections, and stored free floating in the in-house-made antifreeze solution (30% glycerol, 30% ethylene glycol, 20% ddH_20, and 20% PBS 0.1M) at −20°C.

RNA isolation

Total RNA was isolated from the epidermis of two 50-mm sections per subject, after tissue dissecting under the microscope, using TruXtract FFPE total NA kit—column (Covaris, cat. no. PN520220) and PureLink FFPE Total RNA Isolation Kit (Invitrogen, cat. no. K1560-02), according to the manufacturer’s instructions. Both kits are designed for efficient extraction of nucleic acids from fixed tissue samples and resulted in high yields of high-quality RNA well suited for analytical methods such as next-generation sequencing or qPCR/RT-qPCR. The RNA purity and concentrations were measured by NanoDrop ND-1000 Spectrophotometer (Thermo Fisher Scientific) before the preparation of the miRNA array. All RNA samples achieving adequate purity ratios (A260/A280 = 1.7–2.0) were used for subsequent analysis (Table S2).

miRNA profiling

miRNA expression analysis was performed using TaqMan Array Human MicroRNA A + B Cards (Thermo Fisher Scientific) containing 754 miRNAs. Each array includes three TaqMan MicroRNA Assay endogenous controls to aid in data normalization (RNU44-001094 N = 1 per card, RNU48-001006 N = 1 per card, U6 snRNA-001973 N = 4 per card), and one TaqMan MicroRNA Assay not related to humans as a negative control (assay ID 000338, ath-miR-159). An amount of 15 ng of total RNA was reverse transcribed using Megaplex RT Primers, Human Pool A v2.1 and Megaplex and RT Primers and Human Pool B v3.0. cDNA was pre-amplified using Megaplex PreAmp Primers, Human Pool A v2.1 and Megaplex PreAmp Primers, Human Pool B v3.0, respectively, according to the manufacturers’ instructions. The pre-amplification products were diluted in 75 μL of 0.1x TE buffer, pH 8.0, and used for the RT-qPCR reaction. PCR reaction mix was prepared using 9 μL of the diluted pre-amplification product, 450 μL TaqMan Fast Advanced Master Mix, and 441 μL Nuclease-free water. Each reservoir of the card was loaded with 100 μL of the PCR mix and centrifuged. RT-qPCR experiments were performed on the ViiATM 7 Fast Real-Time PCR System (Thermo Fisher Scientific), following the recommended cycling protocol: enzyme activation at 92°C for 10 min, followed by 40 cycles of denaturation at 95°C for 1 s and annealing at 60°C for 20 s. The reaction volume of each micro-well was 1 μL.

Threshold algorithms

The threshold is the level of fluorescence above the baseline and within the exponential growth region of the amplification curve.

The Cq value is the fractional cycle at which an amplification plot crosses the fluorescence threshold.[35] The two definitions are fundamental in every qPCR experiment, however, there are substantial differences in their generation and application. Namely, the number of analyzed targets, samples, and diversity among them, as well as the technology used must be considered in every experiment. There are several possibilities when it comes to threshold setting. The most common procedure of quantification is referred to as the Ct method, or "baseline threshold" method, where the threshold could be manually or automatically set. An alternative method called the C_{RT}, or the "relative threshold" method, has proven to be more robust for analyzing data from microarray data.[24]

The manually fixed threshold is usually applied for a low number of targets. The users can set the log view of generated amplification plots to determine the background-derived signal plots (first cycles) and put the threshold to the closest point where the background signal is not crossing it.

The automatic threshold is set automatically by the instrument, one for each curve/miRNA of the experiment. It is based on the assumption that data exhibit a typical amplification plot with plateau phase, linear phase, exponential or geometric phase, and baseline. The baseline threshold algorithm subtracts a baseline component and sets a fluorescent threshold in the exponential region for miRNA quantification. It is easy to use but it can lead to inadequate quantification if the curves are not in sigmoid shape.

The most recent algorithm, the relative threshold (C_{RT}) method is recommended by the manufacturer. It calculates C_{RT} values for each amplification curve, and no information is needed from the other curves. The amplification curve is first set to a relative scale by setting the minimum relative fluorescence value to 0 and the maximum value to 1. A reaction efficiency curve (model) is created for each amplification curve. A reference efficiency level is used to find the fractional cycle where efficiency curve (model) reaches a specific value. Then the fluorescence level is determined, and the relative fluorescence threshold is calculated. C_{RT} is computed as the fractional cycle where the amplification curve crosses the relative fluorescence threshold. When it comes to array technology allowing the analysis of hundreds of targets, this is the method recommended by ThermoFisher (TaqMan miRNA Array Human MicroRNA A + B Cards Set protocol), since it facilitates analysis of amplifications in low-volume reaction, analysis without passive dye normalization, and high throughput analysis tuned to a high number of reactions. This method takes all of the curves for a particular target into account (assay-based analysis). There is no need to define a baseline for the curves since the C_{RT} algorithm obtains a Cq value that is not dependent on the threshold value. The automatic threshold and C_{RT} methods are based on proprietary algorithms (Thermo Fisher Scientific). In this work, all threshold settings were applied in DataConnect cloud through Design and Analysis software (DA2) (Thermo Fisher Scientific, online version).

Amplification curve QC

As a measure for amplification quality when handling hundreds of amplifications in a single run, the users have at their disposal different parameters that allow automatic quality filtering. The three useful parameters are Amp Status, the AmpScore, and the Cq confidence.

AmpStatus is a categorical result assessing normal amplification behavior and defining three categories: "AMP" if amplification is present, "No-AMP" for the absence, and "inconclusive" for a curve difficult to classify that needs to be reviewed. Since the algorithm uses information from all curves to determine the AmpStatus, it is sensitive to the number of curves. The AmpScore is a continuous metric of reaction quality for amplification curves that can be used for all qPCR applications. It allows automatized checks of amplified vs. non-amplified reactions. This score is very helpful because it reliably substitutes the time-consuming manual inspection of the single amplification curve. It helps to resolve ambivalent data and address false positives and false negatives. The AmpScore algorithm implies that the height of amplification curve linear region correlates with reaction quality where high linear region rise is associated with strong amplification, low linear region rises with weak amplification, and non-existent linear region with non-amplification. Numerically, it ranges between 0 and 2 with values below 1, meaning that amplification does not reach the required quality "threshold" whereas above 1 is considered good. The Cq confidence value is a measure of Cq reliability, answering the question of how reliable is the Cq value obtained, and not whether it has been amplified or not. It ranges from 0 to 1 with values greater than 0.8 (default) considered good and >0.95 very confident. It is measured in the context of the amplification curve itself, and not the relationship with other curves. In this work, we showed how the amplification curve QC is helpful to substitute time-consuming manual inspection.

Endogenous control selection

Many variables can influence the endogenous selection such as the experimental settings, the origin of the tissue sample and its heterogeneity, the quantity and stability in different specimens, and the sample handling and storage. Thus, the stability of reference miRNAs needs to be checked in each experimental condition. To address the variability issue in the presented experimental system, we followed two main strategies using as input only the miRNAs expressed in all samples (call rate 100%): (1) identification of stable endogenous miRNAs according to stability ranks and (2) the calculation geometric mean by card, as a normalization factor. To find the most stable miRNAs, in both cards separately, we used the user-friendly web-based tool RefFinder (https://www.ciidirsinaloa.com.mx/RefFinder-master/ or http://www.heartcure.com.au/reffinder/)[43] developed for evaluating and screening reference genes/miRNAs from extensive experimental datasets. It integrates the most widely used computational programs: geNorm, Normfinder, BestKeeper, and the comparative Delta-Ct method.[44–47] BestKeeper is an Excel-based software tool that evaluates miRNA expression stability by calculating the standard deviation (SD) and coefficient of variation of the Cq values. A smaller SD indicates better stability of gene expression.[46] Normfinder calculates a

stability value by combining intragroup and intergroup variation for candidate reference genes.[45] geNorm calculates the average pairwise variation for a reference with all other miRNAs and presents it as the M value. The lowest M value represents the most stable gene expression.[45] Delta Ct compares the relative expression of pairs of reference miRNAs within each sample.[47] Finally, the recommended comprehensive ranking is calculated by RefFinder, which automatically assigns an appropriate weight to individual miRNAs and calculates the geometric mean of their weights for the overall final ranking, based on the rankings from each program. We selected two different references, one for each card set (A and B), as only the endogenous controls (U6, RNU48, RNU44) set by the manufacturer were present both in cards, allowing us to calibrate each plate individually. Indeed, each card represents a different experiment, each one requiring independent retrotranscription (pool A or B specific), pre-amplification (pool specific), and card loading steps, as well as a different run on the instrument. As second normalization approach, we calculated the geometric mean of Cq values, based on all miRNAs expressed in 100% of samples in each card. Compared with arithmetic mean, it controls better for extreme values and abundance differences between the different miRNAs. Geometric mean cannot be calculated if a set of values contains zero or if they are negative. Geometric mean was calculated using the geometric.mean function as implemented in the R psych package.[48]

Relative expression analysis

Only the miRNAs that were detected in at least 90% (call rate $\geq 90\%$) of the samples were considered. The DE of miRNAs was quantified as RQ via the $2^{-\Delta\Delta ct}$ approach[18] with selected endogenous controls for normalization and healthy control samples used as the reference group. We calculated $\Delta\Delta$Cq as mean ΔCq (miRNA of interest in the group of interest)—mean of ΔCq (miRNA of interest in the reference group). Then, the FC in expression was calculated as $2^{-\Delta\Delta Cq}$. For a reduction of expression in the group of interest with respect to controls we transformed as the negative inverse of $2^{-\Delta\Delta Cq}$ to provide with the FC reduction in expression. Comparisons of miRNA expression values in painful peripheral neuropathy patients and healthy controls were performed according to Wilcoxon rank-sum test. The relationship among results, obtained with different settings, was investigated with a correlation matrix considering FC and p value.

Target annotation and GO enrichment analysis

For individual target analysis of miRNAs present in at least 95% of 19 healthy subjects and to identify genes that represent putative targets, a prediction analysis was performed by DIANA-TarBase v7[49] that provides hundreds of thousands of high-quality manually curated experimentally validated miRNA:gene interactions. We selected genes from experiments in skin tissue. Moreover, ClueGO app (v2.5.8) from Cytoscape 3.9.1[50] was applied to identify enriched GO Biological Processes and Molecular Functions starting from the list of miRNA targets. We performed an over-representation analysis based on an enrichment right-sided hypergeometric test that uses Bonferroni as multiple testing correction. Enriched terms with a p value <0.05 were considered statistically significant and are represented with bar graphs.

DATA AND CODE AVAILABILITY

All data are available in the main text or the supplemental information. The raw data are deposited in the institutional database and are available upon request at https://doi.org/10.5281/zenodo.7589088.

SUPPLEMENTAL INFORMATION

Supplemental information can be found online at https://doi.org/10.1016/j.omtm.2023.101116.

ACKNOWLEDGMENTS

We thank Dr. Rosina Paterra from the Molecular Neuro-Oncology Unit (Fondazione IRCCS Istituto Neurologico Carlo Besta) for advice and technical support with RNA isolation. We thank the technical support of Thermo Fisher Scientific for troubleshooting. The authors also thank Rosalind Hendricks, Assistant Biomedical Librarian of the "Fondazione IRCCS Istituto Neurologico Carlo Besta," for English language revision of our article.

This work was supported with PAIN-net Project: Molecule-to-Man Pain Network, EU Research Framework Programme H2020/Marie Skłodowska-Curie Actions, grant agreement number 721841 and Italian Ministry of Health (RRC), Italy.

AUTHOR CONTRIBUTIONS

M.A. and E.S. conceptualized the study. M.A., M.M., and S.M. designed and M.A. performed the experiments. R.L. processed all specimens. E.S. performed the data analysis. G.L. and C.F. performed supervision and funding acquisition. All the authors wrote and edited the manuscript.

DECLARATION OF INTERESTS

The authors declare no competing interests.

REFERENCES

1. Chakraborty, C., Sharma, A.R., Sharma, G., and Lee, S.S. (2021). Therapeutic advances of miRNAs: A preclinical and clinical update. J. Adv. Res. *28*, 127–138. https://doi.org/10.1016/j.jare.2020.08.012.
2. Condrat, C.E., Thompson, D.C., Barbu, M.G., Bugnar, O.L., Boboc, A., Cretoiu, D., Suciu, N., Cretoiu, S.M., and Voinea, S.C. (2020). miRNAs as Biomarkers in Disease: Latest Findings Regarding Their Role in Diagnosis and Prognosis. Cells *9*, 276. https://doi.org/10.3390/cells9020276.
3. Garofalo, M., Condorelli, G., and Croce, C.M. (2008). MicroRNAs in diseases and drug response. Curr. Opin. Pharmacol. *8*, 661–667. https://doi.org/10.1016/j.coph.2008.06.005.
4. Hanna, J., Hossain, G.S., and Kocerha, J. (2019). The Potential for microRNA Therapeutics and Clinical Research. Front. Genet. *10*, 478. https://doi.org/10.3389/fgene.2019.00478.
5. Merritt, C.R., Ong, G.T., Church, S.E., Barker, K., Danaher, P., Geiss, G., Hoang, M., Jung, J., Liang, Y., McKay-Fleisch, J., et al. (2020). Multiplex digital spatial profiling of proteins and RNA in fixed tissue. Nat. Biotechnol. *38*, 586–599. https://doi.org/10.1038/s41587-020-0472-9.
6. Amatori, S., and Fanelli, M. (2022). The Current State of Chromatin Immunoprecipitation (ChIP) from FFPE Tissues. Int. J. Mol. Sci. *23*, 1103. https://doi.org/10.3390/ijms23031103.
7. Yadav, R.P., Polavarapu, V.K., Xing, P., and Chen, X. (2022). FFPE-ATAC: A Highly Sensitive Method for Profiling Chromatin Accessibility in Formalin-Fixed Paraffin-Embedded Samples. Curr. Protoc. *2*, e535. https://doi.org/10.1002/cpz1.535.

8. Hedegaard, J., Thorsen, K., Lund, M.K., Hein, A.M.K., Hamilton-Dutoit, S.J., Vang, S., Nordentoft, I., Birkenkamp-Demtröder, K., Kruhøffer, M., Hager, H., et al. (2014). Next-generation sequencing of RNA and DNA isolated from paired fresh-frozen and formalin-fixed paraffin-embedded samples of human cancer and normal tissue. PLoS One *9*, e98187. https://doi.org/10.1371/journal.pone.0098187.

9. Cazzato, D., and Lauria, G. (2017). Small fibre neuropathy. Curr. Opin. Neurol. *30*, 490–499. https://doi.org/10.1097/WCO.0000000000000472.

10. Devigili, G., Cazzato, D., and Lauria, G. (2020). Clinical diagnosis and management of small fiber neuropathy: an update on best practice. Expert Rev. Neurother. *20*, 967–980. https://doi.org/10.1080/14737175.2020.1794825.

11. Lauria, G., and Devigili, G. (2007). Skin biopsy as a diagnostic tool in peripheral neuropathy. Nat. Clin. Pract. Neurol. *3*, 546–557. https://doi.org/10.1038/ncpneuro0630.

12. Lauria, G., Faber, C.G., and Cornblath, D.R. (2022). Skin biopsy and small fibre neuropathies: facts and thoughts 30 years later. J. Neurol. Neurosurg. Psychiatry *93*, 915–918. https://doi.org/10.1136/jnnp-2021-327742.

13. Baron, R., Dickenson, A.H., Calvo, M., Dib-Hajj, S.D., and Bennett, D.L. (2023). Maximizing treatment efficacy through patient stratification in neuropathic pain trials. Nat. Rev. Neurol. *19*, 53–64. https://doi.org/10.1038/s41582-022-00741-7.

14. Baron, R., Binder, A., and Wasner, G. (2010). Neuropathic pain: diagnosis, pathophysiological mechanisms, and treatment. Lancet Neurol. *9*, 807–819. https://doi.org/10.1016/S1474-4422(10)70143-5.

15. Saliminejad, K., Khorram Khorshid, H.R., and Ghaffari, S.H. (2019). Why have microRNA biomarkers not been translated from bench to clinic? Future Oncol. *15*, 801–803. https://doi.org/10.2217/fon-2018-0812.

16. Wang, B., and Xi, Y. (2013). Challenges for MicroRNA Microarray Data Analysis. Microarrays *2*, 34–50. https://doi.org/10.3390/microarrays2020034.

17. Pritchard, C.C., Cheng, H.H., and Tewari, M. (2012). MicroRNA profiling: approaches and considerations. Nat. Rev. Genet. *13*, 358–369. https://doi.org/10.1038/nrg3198.

18. Livak, K.J., and Schmittgen, T.D. (2001). Analysis of relative gene expression data using real-time quantitative PCR and the 2(-Delta Delta C(T)) Method. Methods *25*, 402–408. https://doi.org/10.1006/meth.2001.1262.

19. Schmittgen, T.D., and Livak, K.J. (2008). Analyzing real-time PCR data by the comparative C(T) method. Nat. Protoc. *3*, 1101–1108. https://doi.org/10.1038/nprot.2008.73.

20. Kozera, B., and Rapacz, M. (2013). Reference genes in real-time PCR. J. Appl. Genet. *54*, 391–406. https://doi.org/10.1007/s13353-013-0173-x.

21. Drobna, M., Szarzyńska-Zawadzka, B., Daca-Roszak, P., Kosmalska, M., Jaksik, R., Witt, M., and Dawidowska, M. (2018). Identification of Endogenous Control miRNAs for RT-qPCR in T-Cell Acute Lymphoblastic Leukemia. Int. J. Mol. Sci. *19*, 2858. https://doi.org/10.3390/ijms19102858.

22. Kaur, J., Saul, D., Doolittle, M.L., Rowsey, J.L., Vos, S.J., Farr, J.N., Khosla, S., and Monroe, D.G. (2022). Identification of a suitable endogenous control miRNA in bone aging and senescence. Gene *835*, 146642. https://doi.org/10.1016/j.gene.2022.146642.

23. Muñoz, J.J., Anauate, A.C., Amaral, A.G., Ferreira, F.M., Meca, R., Ormanji, M.S., Boim, M.A., Onuchic, L.F., and Heilberg, I.P. (2020). Identification of housekeeping genes for microRNA expression analysis in kidney tissues of Pkd1 deficient mouse models. Sci. Rep. *10*, 231. https://doi.org/10.1038/s41598-019-57112-4.

24. Applied Biosystems. Crt, a relative threshold method for qPCR data analysis on the QuantStudio 12K Flex system with OpenArray technology. https://assets.thermofisher.com/TFS-Assets/LSG/brochures/CO28730-Crt-Tech-note_FLR.pdf.

25. Martinelli-Boneschi, F., Colombi, M., Castori, M., Devigili, G., Eleopra, R., Malik, R.A., Ritelli, M., Zoppi, N., Dordoni, C., Sorosina, M., et al. (2017). COL6A5 variants in familial neuropathic chronic itch. Brain *140*, 555–567. https://doi.org/10.1093/brain/aww343.

26. Korfitis, C., Gregoriou, S., Antoniou, C., Katsambas, A.D., and Rigopoulos, D. (2014). Skin biopsy in the context of dermatological diagnosis: a retrospective cohort study. Dermatol. Res. Pract. *2014*, 734906. https://doi.org/10.1155/2014/734906.

27. Newton, Y., Sedgewick, A.J., Cisneros, L., Golovato, J., Johnson, M., Szeto, C.W., Rabizadeh, S., Sanborn, J.Z., Benz, S.C., and Vaske, C. (2020). Large scale, robust, and accurate whole transcriptome profiling from clinical formalin-fixed paraffin-embedded samples. Sci. Rep. *10*, 17597. https://doi.org/10.1038/s41598-020-74483-1.

28. Pennock, N.D., Jindal, S., Horton, W., Sun, D., Narasimhan, J., Carbone, L., Fei, S.S., Searles, R., Harrington, C.A., Burchard, J., et al. (2019). RNA-seq from archival FFPE breast cancer samples: molecular pathway fidelity and novel discovery. BMC Med. Genomics *12*, 195. https://doi.org/10.1186/s12920-019-0643-z.

29. Bustin, S., and Nolan, T. (2017). Talking the talk, but not walking the walk: RT-qPCR as a paradigm for the lack of reproducibility in molecular research. Eur. J. Clin. Invest. *47*, 756–774. https://doi.org/10.1111/eci.12801.

30. Gödecke, A. (2018). qPCR-25 years old but still a matter of debate. Cardiovasc. Res. *114*, 201–202. https://doi.org/10.1093/cvr/cvx220.

31. Lou, G., Ma, N., Xu, Y., Jiang, L., Yang, J., Wang, C., Jiao, Y., and Gao, X. (2015). Differential distribution of U6 (RNU6-1) expression in human carcinoma tissues demonstrates the requirement for caution in the internal control gene selection for microRNA quantification. Int. J. Mol. Med. *36*, 1400–1408. https://doi.org/10.3892/ijmm.2015.2338.

32. Shen, J., Wang, Q., Gurvich, I., Remotti, H., and Santella, R.M. (2016). Evaluating normalization approaches for the better identification of aberrant microRNAs associated with hepatocellular carcinoma. Hepatoma Res. *2*, 305–315. https://doi.org/10.20517/2394-5079.2016.28.

33. Lamba, V., Ghodke-Puranik, Y., Guan, W., and Lamba, J.K. (2014). Identification of suitable reference genes for hepatic microRNA quantitation. BMC Res. Notes *7*, 129. https://doi.org/10.1186/1756-0500-7-129.

34. Taylor, S.C., Nadeau, K., Abbasi, M., Lachance, C., Nguyen, M., and Fenrich, J. (2019). The Ultimate qPCR Experiment: Producing Publication Quality, Reproducible Data the First Time. Trends Biotechnol. *37*, 761–774. https://doi.org/10.1016/j.tibtech.2018.12.002.

35. Bustin, S.A., Benes, V., Garson, J.A., Hellemans, J., Huggett, J., Kubista, M., Mueller, R., Nolan, T., Pfaffl, M.W., Shipley, G.L., et al. (2009). The MIQE guidelines: minimum information for publication of quantitative real-time PCR experiments. Clin. Chem. *55*, 611–622. https://doi.org/10.1373/clinchem.2008.112797.

36. Le Carré, J., Lamon, S., and Léger, B. (2014). Validation of a multiplex reverse transcription and pre-amplification method using TaqMan(®) MicroRNA assays. Front. Genet. *5*, 413. https://doi.org/10.3389/fgene.2014.00413.

37. Chen, C., Ridzon, D.A., Broomer, A.J., Zhou, Z., Lee, D.H., Nguyen, J.T., Barbisin, M., Xu, N.L., Mahuvakar, V.R., Andersen, M.R., et al. (2005). Real-time quantification of microRNAs by stem-loop RT-PCR. Nucleic Acids Res. *33*, e179. https://doi.org/10.1093/nar/gni178.

38. Applied Biosystems. TaqMan Advanced miRNA Assays—superior performance for miRNA detection and quantification. https://assets.thermofisher.com/TFS-Assets/GSD/Technical-Notes/TaqMan-Advanced-miRNA-Performance-White-Paper.pdf.

39. Li, J., Smyth, P., Flavin, R., Cahill, S., Denning, K., Aherne, S., Guenther, S.M., O'Leary, J.J., and Sheils, O. (2007). Comparison of miRNA expression patterns using total RNA extracted from matched samples of formalin-fixed paraffin-embedded (FFPE) cells and snap frozen cells. BMC Biotechnol. *7*, 36. https://doi.org/10.1186/1472-6750-7-36.

40. Zhao, Y., Mehta, M., Walton, A., Talsania, K., Levin, Y., Shetty, J., Gillanders, E.M., Tran, B., and Carrick, D.M. (2019). Robustness of RNA sequencing on older formalin-fixed paraffin-embedded tissue from high-grade ovarian serous adenocarcinomas. PLoS One *14*, e0216050. https://doi.org/10.1371/journal.pone.0216050.

41. Xi, Y., Nakajima, G., Gavin, E., Morris, C.G., Kudo, K., Hayashi, K., and Ju, J. (2007). Systematic analysis of microRNA expression of RNA extracted from fresh frozen and formalin-fixed paraffin-embedded samples. RNA *13*, 1668–1674. https://doi.org/10.1261/rna.642907.

42. Lauria, G., Bakkers, M., Schmitz, C., Lombardi, R., Penza, P., Devigili, G., Smith, A.G., Hsieh, S.T., Mellgren, S.I., Umapathi, T., et al. (2010). Intraepidermal nerve fiber density at the distal leg: a worldwide normative reference study. J. Peripher. Nerv. Syst. *15*, 202–207. https://doi.org/10.1111/j.1529-8027.2010.00271.x.

43. Xie, F., Wang, J., and Zhang, B. (2023). RefFinder: a web-based tool for comprehensively analyzing and identifying reference genes. Funct. Integr. Genomics *23*, 125. https://doi.org/10.1007/s10142-023-01055-7.

44. Vandesompele, J., De Preter, K., Pattyn, F., Poppe, B., Van Roy, N., De Paepe, A., and Speleman, F. (2002). Accurate normalization of real-time quantitative RT-PCR data by geometric averaging of multiple internal control genes. Genome Biol. *3*. RESEARCH0034. https://doi.org/10.1186/gb-2002-3-7-research0034.

45. Andersen, C.L., Jensen, J.L., and Ørntoft, T.F. (2004). Normalization of real-time quantitative reverse transcription-PCR data: a model-based variance estimation approach to identify genes suited for normalization, applied to bladder and colon cancer data sets. Cancer Res. *64*, 5245–5250. https://doi.org/10.1158/0008-5472.CAN-04-0496.

46. Pfaffl, M.W., Tichopad, A., Prgomet, C., and Neuvians, T.P. (2004). Determination of stable housekeeping genes, differentially regulated target genes and sample integrity: BestKeeper–Excel-based tool using pair-wise correlations. Biotechnol. Lett. *26*, 509–515. https://doi.org/10.1023/b:bile.0000019559.84305.47.

47. Silver, N., Best, S., Jiang, J., and Thein, S.L. (2006). Selection of housekeeping genes for gene expression studies in human reticulocytes using real-time PCR. BMC Mol. Biol. *7*, 33. https://doi.org/10.1186/1471-2199-7-33.

48. W, R. (2022). Psych: Procedures for Psychological, Psychometric, and Personality Research (Northwestern University). R package version 2.2.9.

49. Vlachos, I.S., Paraskevopoulou, M.D., Karagkouni, D., Georgakilas, G., Vergoulis, T., Kanellos, I., Anastasopoulos, I.L., Maniou, S., Karathanou, K., Kalfakakou, D., et al. (2015). DIANA-TarBase v7.0: indexing more than half a million experimentally supported miRNA:mRNA interactions. Nucleic Acids Res. *43*, D153–D159. https://doi.org/10.1093/nar/gku1215.

50. Shannon, P., Markiel, A., Ozier, O., Baliga, N.S., Wang, J.T., Ramage, D., Amin, N., Schwikowski, B., and Ideker, T. (2003). Cytoscape: a software environment for integrated models of biomolecular interaction networks. Genome Res. *13*, 2498–2504. https://doi.org/10.1101/gr.1239303.

OMTM, Volume *31*

Supplemental information

Archival skin biopsy specimens as a tool for miRNA-based diagnosis: Technical and post-analytical considerations

Mirna Andelic, Margherita Marchi, Stefania Marcuzzo, Raffaella Lombardi, Catharina G. Faber, Giuseppe Lauria, and Erika Salvi

Table S1. Demographic characteristics of the study groups.

	Patients (n=31)	HC (n=19)
**Female**	15 (48.39%)	9 (47.37%)
**Age at biopsy**	48.9±16.3	44.7±16.9

Age and sex are not statistically significantly different between groups.

Table S2. RNA purity and concentrations values measured by NanoDrop ND-1000 Spectrophotometer.

Sample ID	Phenotype	RNA QC (NanoDrop)	
		c (ng/μl)	Purity (260/280)
1132	Pain	6.5	1.81
2048	Pain	5.02	1.90
2137	Pain	6.06	1.87
2453	Pain	8.09	1.90
2511	HC	6.69	1.80
2614	HC	7.93	1.92
2696	Pain	7.70	1.80
2780	HC	5.0	1.85
2841	Pain	8.50	1.80
2856	Pain	8.20	1.95
2913	Pain	8.16	1.99
2971	Pain	8.65	1.90
3077	HC	13.56	1.82
3105	HC	5.0	1.92
3106	HC	5.3	2.00
3125	HC	5.6	1.85
3258	Pain	7.93	1.80
3269	HC	9.93	1.87
3288	Pain	8.06	1.80
3508	HC	8.5	2.0
874	Pain	8.1	2.00
978	HC	7.7	1.93
HC_1	HC	12.20	1.80
HC_2	HC	7.00	1.92
HC_3	HC	13.50	1.85
HC_4	HC	5.1	1.80
HC_5	HC	5.20	1.80
HC_6	HC	4.85	1.82

Sample ID	Phenotype	RNA QC (NanoDrop)	
		c (ng/µl)	Purity (260/280)
HC_7	HC	4.89	1.80
HC_8	HC	6.40	1.94
HC_9	HC	7.70	1.83
M1	Pain	8.10	1.85
M2	Pain	5.50	1.90
M3	Pain	9.70	1.90
M4	Pain	4.50	1.85
M5	Pain	5.20	1.78
M6	Pain	9.50	1.79
SFN_1	Pain	13.40	1.80
SFN_11	Pain	7.30	2.01
SFN_12	Pain	7.50	1.85
SFN_13	Pain	5.00	1.82
SFN_14	Pain	6.70	1.80
SFN_2	Pain	8.80	1.82
SFN_3	Pain	7.10	1.80
SFN_4	Pain	7.00	2.00
SFN_5	Pain	8.80	1.92
SFN_6	Pain	5.10	1.90
SFN_7	Pain	4.20	1.80
SFN_8	Pain	7.30	1.90
SFN_9	Pain	5.10	1.93

Table S3. Comparison results of Cq values among Automatic, C_{RT} and fixed thresholds. Kruskal-Wallis rank sum test with Dunn's test post-hoc analysis was used for multiple comparisons. Post-hoc analysis results were shown only for significant Kruskal Wallis p-values. Only good amplified miRNAs with call rate>90 and median Cq>32 were compared.

Table S4. Expression stability values of miRNAs in all samples applying BestKeeper, delta Ct, geNorm, Normfinder, and RefFinder algorithms, using C_{RT} threshold. MiRNA expressed in 100% of samples (CR=100) were considered. Abbreviations: SD standard deviation, MV M-value, SV stability value, GM geometric mean.

		BestKeeper		Delta CT		Genorm		Normfinder		RefFinder	
**Card**	**miRNA**	**Rank**	**SD**	**Rank**	**SD**	**Rank**	**MV**	**Rank**	**SV**	**Rank**	**GM**
	**miR-200c-002300**	11	1.54	1	1.02	6	0.73	1	0.38	1	2.96
	miR-193b-002367	9	1.45	1	1.02	3	0.61	3	0.42	2	3.83
	[illegible]										
	miR-484-001821	6	1.33	6	1.18	1	0.40	12	0.79	4	4.90
	miR-574-3p-002349	3	1.27	15	1.30	1	0.40	25	0.97	5	6.44
	let-7b-002619	5	1.32	7	1.19	5	0.70	8	0.75	6	6.82
	miR-320-002277	4	1.28	8	1.20	4	0.66	11	0.79	7	7.17
	miR-139-5p-002289	7	1.34	5	1.16	7	0.76	7	0.70	8	7.24
card A	miR-197-000497	2	1.23	14	1.29	2	0.49	23	0.96	9	7.34
	let-7c-000379	13	1.59	3	1.14	9	0.79	6	0.67	10	7.47
	miR-222-002276	20	1.71	4	1.15	21	1.01	5	0.66	11	10.61
	miR-30c-000419	25	1.84	4	1.15	22	1.02	4	0.65	12	11.25
	miR-191-002299	16	1.62	7	1.19	10	0.81	10	0.76	13	11.52
	let-7d-002283	17	1.64	9	1.23	13	0.88	15	0.82	14	14.68
	U6	1	0.82	26	1.63	36	1.24	38	1.38	15	15.20
	miR-24-000402	21	1.75	8	1.20	25	1.06	9	0.75	16	15.93
	miR-328-000543	14	1.60	13	1.27	14	0.90	20	0.91	17	16.81

		BestKeeper		Delta CT		Genorm		Normfinder		RefFinder	
**Card**	**miRNA**	**Rank**	**SD**	**Rank**	**SD**	**Rank**	**MV**	**Rank**	**SV**	**Rank**	**GM**
	miR-146a-000468	19	1.67	9	1.23	26	1.07	16	0.83	18	18.41
	miR-331-000545	26	1.87	11	1.24	23	1.03	13	0.80	19	19.67
	miR-365-001020	30	1.99	10	1.23	20	0.99	14	0.80	20	19.68
	miR-125a-5p-002198	20	1.71	16	1.32	12	0.86	22	0.95	21	19.71
	miR-342-3p-002260	15	1.61	19	1.37	11	0.84	31	1.07	22	20.06
	miR-211-000514	12	1.55	19	1.37	17	0.94	28	1.03	23	20.64
	miR-145-002278	20	1.71	15	1.30	16	0.93	21	0.92	24	20.84
	miR-150-000473	18	1.66	17	1.35	15	0.91	26	1.01	25	21.01
	miR-223-002295	24	1.80	12	1.25	27	1.08	17	0.83	26	21.82
	miR-99b-000436	28	1.93	13	1.27	19	0.98	19	0.89	27	22.21
	miR-100-000437	29	1.98	12	1.25	24	1.04	18	0.84	28	22.74
	miR-92a-000431	22	1.76	17	1.35	18	0.97	27	1.02	29	24.03
	miR-345-002186	19	1.67	18	1.36	28	1.10	24	0.97	30	25.06
	miR-483-5p-002338	8	1.44	27	1.65	37	1.26	39	1.38	31	26.08
	miR-146b-001097	19	1.67	22	1.43	29	1.11	33	1.10	32	28.43
	miR-126-002228	27	1.88	19	1.37	31	1.14	29	1.03	33	29.96
	miR-186-002285	23	1.77	21	1.42	30	1.13	32	1.08	34	30.43
	miR-203-000507	30	1.99	20	1.38	32	1.16	30	1.05	35	32.19
	miR-19b-000396	31	2.06	23	1.49	33	1.18	34	1.20	36	34.49
	miR-195-000494	32	2.23	24	1.52	34	1.20	35	1.24	37	35.49
	miR-29c-000587	33	2.28	24	1.52	35	1.22	36	1.25	38	36.49
	miR-27a-000408	35	2.37	25	1.61	38	1.28	37	1.37	39	38.23
	miR-125b-000449	36	2.38	28	1.66	39	1.30	40	1.41	40	40.25
	miR-374-000563	34	2.33	29	1.79	40	1.32	41	1.59	41	40.49
card B	**hsa-miR-99b#-002196**	1	0.82	1	1.18	1	0.825	1	0.657	1	1
	hsa-miR-338-5P-002658	2	0.91	4	1.31	1	0.825	4	0.915	2	2.38
	hsa-miR-1285-002822	4	1.04	2	1.2	3	1.049	2	0.668	3	2.83
	hsa-miR-378-002243	5	1.2	3	1.3	2	0.968	3	0.853	4	3.41
	U6	3	0.96	6	1.39	4	1.137	6	1.043	5	4.82
	hsa-miR-1201-002781	6	1.4	5	1.38	5	1.206	5	0.985	6	5.48
	hsa-miR-30d-000420	7	2.11	7	1.72	6	1.353	7	1.501	7	7

Table S5. Stability ranking obtained with the recommended comprehensive analysis from RefFinder, applying different threshold settings. MiRNA expressed in 100% of samples (CR=100) were considered.

	Pool A			Pool B		
**Ranking**	**Fixed**	**Automatic**	**CRT**	**Fixed**	**Automatic**	**CRT**
**1**	**miR-193b-002367**	**miR-200c-002300**	**miR-200c-002300**	**U6-snRNA-001973**	**miR-99b#-002196**	**miR-99b#-002196**
**2**	miR-200c-002300	miR-200b-002251	miR-193b-002367	miR-1285-002822	miR-1285-002822	miR-338-5P-002658
**3**	let-7b-002619	let-7b-002619	miR-200b-002251	miR-99b#-002196	miR-338-5P-002658	miR-1285-002822
**4**	miR-320-002277	miR-193b-002367	miR-484-001821	miR-338-5P-002658	miR-378-002243	miR-378-002243
**5**	let-7c-000379	miR-484-001821	miR-574-3p-002349	miR-378-002243	U6-snRNA-001973	U6-snRNA-001973
**6**	miR-200b-002251	miR-574-3p-002349	let-7b-002619	miR-1201-002781	miR-1201-002781	miR-1201-002781
**7**	miR-191-002299	miR-197-000497	miR-320-002277		miR-30d-000420	miR-30d-000420
**8**	miR-328-000543	miR-320-002277	miR-139-5p-002289			
**9**	miR-222-002276	let-7c-000379	miR-197-000497			
**10**	miR-92a-000431	miR-222-002276	let-7c-000379			
**11**	miR-484-001821	miR-30c-000419	miR-222-002276			
**12**	let-7d-002283	miR-139-5p-002289	miR-30c-000419			
**13**	miR-139-5p-002289	miR-191-002299	miR-191-002299			
**14**	miR-30c-000419	miR-328-000543	let-7d-002283			
**15**	miR-331-000545	miR-24-000402	U6-snRNA-001973			
**16**	U6-snRNA-001973	miR-331-000545	miR-24-000402			
**17**	miR-211-000514	let-7d-002283	miR-328-000543			
**18**	miR-24-000402	U6-snRNA-001973	miR-146a-000468			
**19**	miR-146a-000468	miR-146a-000468	miR-331-000545			
**20**	miR-574-3p-002349	miR-223-002295	miR-365-001020			
**21**	miR-197-000497	miR-99b-000436	miR-125a-5p-002198			
**22**	miR-223-002295	miR-365-001020	miR-342-3p-002260			
**23**	miR-365-001020	miR-100-000437	miR-211-000514			
**24**	miR-342-3p-002260	miR-211-000514	miR-145-002278			
**25**	miR-99b-000436	miR-345-002186	miR-150-000473			
**26**	miR-483-5p-002338	miR-145-002278	miR-223-002295			
**27**	miR-145-002278	miR-125a-5p-002198	miR-99b-000436			
**28**	miR-125a-5p-002198	miR-92a-000431	miR-100-000437			
**29**	miR-146b-001097	miR-150-000473	miR-92a-000431			
**30**	miR-126-002228	miR-146b-001097	miR-345-002186			
**31**	miR-186-002285	miR-483-5p-002338	miR-483-5p-002338			
**32**	miR-195-000494	miR-342-3p-002260	miR-146b-001097			

	Pool A			Pool B		
**Ranking**	**Fixed**	**Automatic**	**C_{RT}**	**Fixed**	**Automatic**	**C_{RT}**
**33**	miR-150-000473	miR-186-002285	miR-126-002228			
**34**	miR-345-002186	miR-126-002228	miR-186-002285			
**35**	miR-100-000437	miR-203-000507	miR-203-000507			
**36**	miR-19b-000396	miR-195-000494	miR-19b-000396			
**37**	miR-203-000507	miR-19b-000396	miR-195-000494			
**38**	miR-29c-000587	miR-19a-000395	miR-29c-000587			
**39**	miR-374-000563	miR-29c-000587	miR-27a-000408			
**40**		miR-27a-000408	miR-125b-000449			
**41**		miR-125b-000449	miR-374-000563			
**42**		miR-374-000563				

Table S6. miRNA relative expression results comparing painful neuropathic against control group, considering C_{RT}, baseline automatic and manually set datasets. miRNAs are ranked for C_{RT} P-value. The comparisons are made applying Wilcoxon rank sum test. For C_{RT} and baseline automatic, normalization was done using miR-200c-002300 (pool A) and miR-99b#-002196 (pool B) whereas for manually set threshold, hsa-miR-193b-002367 (poolA) and U6-snRNA-001973 (poolB) were used. We compared quality-controlled miRNAs with call rate>90 and median Cq>32, in common among the datasets.

Table S7. List of miRNAs expressed in human epidermis from fixed skin biopsy in 19 healthy controls.

Table S8. ClueGO Enrichment analysis results. Molecular functions (MF) and biological processes (BP) inferred from the list of epidermal miRNAs-targets are listed. Bonferroni Pvalue: p value of the GO term after Bonferroni correction. % Associated Genes: percentage of genes found from the total number of associated genes. N Genes: Number of genes from our list of miRNA-targets associated with the GO term. Associated genes are represented from among those associated with GO terms.
